# Asymptomatic viral infection is associated with lower host reproductive output in wild mink populations

**DOI:** 10.1038/s41598-023-36581-8

**Published:** 2023-06-09

**Authors:** Andrzej Zalewski, Jenni M. E. Virtanen, Hanna Zalewska, Tarja Sironen, Marta Kołodziej-Sobocińska

**Affiliations:** 1 Mammal Research Institute, Polish Academy of Sciences, 17-230 Białowieża, Poland; 2 Department of Veterinary Biosciences, Faculty of Veterinary Medicine, University of Helsinki, Agnes Sjöbergin Katu 2, 00790 Helsinki, Finland; 3 Department of Virology, Faculty of Medicine, University of Helsinki, Haartmaninkatu 3, 00290 Helsinki, Finland

**Keywords:** Ecology, Environmental sciences, Natural hazards, Diseases

## Abstract

Many endemic viruses circulate in populations without hosts showing visible signs of disease, while still having the potential to alter host survival or reproduction. Aleutian Mink Disease Virus (AMDV) circulates in many American mink (*Neogale vison*) populations in its native and introduced ranges. In this study, we analysed how AMDV infection in female American mink affects the reproduction of a feral population. Females infected with AMDV delivered significantly smaller litters (5.8 pups) than uninfected females (6.3 pups), meaning their litter size was reduced by 8%. Larger females and yearling females had larger litters than smaller and older females. There were no significant differences in whole litter survival between infected and uninfected females; however, offspring survival until September or October within litters of infected females was 14% lower than that within those of uninfected females. This negative link between infection and reproductive output means that Aleutian disease could seriously affect the wild mink population. This study increases our understanding of the threats posed by the spread of viruses to wildlife from farm animals or humans, highlighting that viruses circulating in wildlife, even in the absence of clinical manifestation, can be important drivers of population dynamics in wildlife.

## Introduction

Diseases have significant ramifications for wildlife population density, distribution, genetic diversity loss, and in consequence, for conservation. Emerging diseases can cause local extirpations and, in extreme cases, even the extinction of species^1^. Viruses are a pathogen that, within a short time, can cause a disease outbreak leading to decreased population densities through mass mortality. Thus, viral disease outbreaks generate great interest and are frequently well described in the literature^2^, e.g. the mass seal mortality caused by phocine distemper virus^3^. Disease outbreaks can affect many different species at various trophic levels, which can significantly alter ecosystem structure (e.g., rabies affects both top predators, such as wolves, and mesopredators, like foxes). Many endemic viruses circulate in populations without visible signs of disease in the hosts^4^, while still having a biologically significant effect on the host. Although such virus strains have the potential to alter host fitness, host population size may not decrease in the short term due to a compensatory response in the non-infected part of the population. In the long term, however, through reduced reproduction or increased mortality, endemic viruses can significantly reduce host abundances^5^. Yet few studies have explored how non-lethal viral infection affects the fitness, and particularly the survival and/or reproduction, of wildlife hosts.

According to the ‘principle of allocation’ of time and energy^6^, the energetic cost of the immune response to a viral infection can compete with other life-history events, such as reproduction, for limited body resources^7^. Therefore, the cost of the immune response may cause infected females to have smaller litters. Indeed, studies have described the effects of viral infection on reproductive output in farmed mammals^8^, but little is known about its impact on wildlife species^9^. Studying the effect of a virus on reproduction is challenging as a number of factors affecting reproduction in wildlife can mask the subtle effects of viral infection. The mother’s traits, such as her size, age or body condition, are important factors affecting reproductive output. Larger females generally give birth to larger litters^10^, and younger females tend to be less productive than older ones (terminal investment hypothesis)^11,12^. However, reproduction can also decrease in older females (reproductive senescence’ hypothesis^13^) or increase with increasing breeding ability and experience (constraint hypothesis)^14^. Moreover, spatial and temporal variation in food abundance or intraspecific competition (population density) can also affect reproductive output, especially in income breeders, for whom reproduction is related to the use of current energetic income^15^. Finally, different methods used to determine litter-size can give different measures of reproductive output, even within single individuals, as different methods usually take measurements at different phases of the reproductive-development process^16^. Generally, counting embryos gives higher litter size estimates than counting placental scars (see the detailed explanation in the “Materials and methods” section)^16^. Thus, these parameters (the mother’s traits and method used) should accounted for when quantifying the impact of a female’s infection status on litter size.

Litter size is only one aspect of reproductive performance; another is the survival of pups to independent life (maturity), which can be affected by an interaction between the mother’s body condition and her health status^17^. Data on pup survival are limited for some mammals, especially those with elusive lifestyles, and are primarily available for larger mammals inhabiting open habitats^12,18,19^. This is related to the relative ease of studying pup survival in such species through field observations of radio-collared females with pups^20^. In our study, we used an indirect method to estimate pup survival relative to the infection status of females. To this end, we analysed inferred pedigree relationships between the females and sets of caught subadult individuals. Inferring pedigree relationships based on the availability of genetic markers like microsatellite loci and single nucleotide polymorphisms can supplement traditional methods of studying mammal reproduction^21^. This approach does not directly measure the survival rate of offspring, as it is not possible to catch all the surviving offspring, particularly as they can disperse prior to catching. However, assuming that the proportion of uncaptured offspring is the same for litters of virus-infected and uninfected females, it is possible to assess the effect of female health status on offspring survival.

The American mink (*Neogale vison*), a medium-sized carnivore, is an invasive species originating from North America that has been introduced to Europe, Asia, and South America^22^. In Europe, the mink was introduced to the eastern and western parts of the continent for different reasons and in different ways. It was purposefully introduced to the former Soviet Union via the release of more than 20,000 American mink at more than 200 sites between 1930 and the 1970s^23^. In the following years mink populations expanded throughout central Europe. In western and northern Europe, the species was imported for breeding on fur farms in the late 1920s. The accidental escape of mink from farms led to the establishing of feral populations in numerous locations across Europe that gradually expanded their range. The American mink range in Europe is still expanding^24^.

After the American mink was brought to Europe, the Aleutian Mink Disease Virus (AMDV) was initially transmitted to ranch-raised individuals in 1946^25^, and later to feral mink via farm escapees. AMDV is a highly contagious parvovirus belonging to the genus *Parvovirus*, family *Parvoviridae*^26^. Because of their small genome size, parvoviruses use an extensive repertoire of genetic strategies to enhance their coding and diversity potential^26^. There is substantial knowledge on the effects of AMDV on fur farmed mink^8,27–29^. Aleutian disease (AD) is often fatal to mink and causes multiple clinical syndromes, such as acute interstitial pneumonia, plasmacytosis, hypergammaglobulinemia, and immune complex-mediated glomerulonephritis, and arteritis^28,30^. Affected mink can display a wide range of clinical signs, including weight loss, poor coat condition, organ dysfunction and neurological abnormalities^28^. The virulence of the virus strain, which can range from non-virulent to extremely virulent^28^, alongside host factors such as age and genotype determine the severity of the symptoms, which can range from subclinical to fatal^26,31^. In addition to direct mortality in adults, AMDV infections can lead to a decrease in fertility and spontaneous abortions^29,32^. Furthermore, AMDV infection in neonatal mink causes acute, rapidly progressing interstitial pneumonia with a high mortality rate, which varies between 30 and 100% depending on the virus strain^33^. AMDV can persist in mink tissues and only up to a quarter of mink have been estimated to clear the virus completely^34^. However, the viremia in blood, feces and mouth can be transient^35,36^.

AMDV has been reported in ranch and feral mink in many regions of North America, Asia and Europe^37–41^. The prevalence of AMDV in wild (from North America) and feral (from Europe) populations has been found to vary between regions, reaching up to 92%^37,39^, and in some regions to fluctuate over time with a peak in prevalence every 3–4 years^37^. AMDV has also been reported in many other carnivore species in North America and Europe, especially in various Mustelids (in 10 species), but also in some Procyonids, Mephitids or Felids^38,42–46^. The virus has also spilled over to endangered species, like the European mink (*Mustela luterola*), potentially accelerating decreases in its densities^42^. Feral mink infected with AMDV had a lower body condition index, and enlarged spleens, livers and kidneys, which potentially indicates that AMDV reduces mink fitness^37^. However, despite the wild spread of AMDV among carnivores and its high prevalence, there is little evidence that AMDV has any detrimental effect on the fitness of feral American mink or other carnivores, particularly on their survival or reproduction.

The aim of our study was to analyse how AMDV infection affects the reproduction of feral American mink. We expected that AMDV infection would reduce mink reproductive output through reducing (a) the number of pups per female (litter size), (b) the number of females for whom at least one pup survived more than 4 months (an indicator of mortality of the whole litter–litter survival), and (c) the proportion of pups that survived more than 4 months (pup survival). We analysed litter and pup survival separately as these measures may be indicative of different effects of the virus on reproduction. Aleutian disease can cause infected females to fail to give birth to live young or their young to die soon after delivery, as is frequently observed on farms. It can also reduce mink pup survival after the young are directly infected by the virus, or potentially when infected females provision the pups with less food. To account for the mothers’ traits affecting reproduction, we also included the size and age of the female in the analyses.

## Materials and methods

### Mink collection and dissection

We collected 1187 feral mink carcasses (717 males and 470 females) at 4 national parks from two regions: eastern (Biebrza National Park—BNP, and Narew National Park—NNP), and western Poland (Drawa National Park—DNP and Warta Mouth National Park—WMNP). These national parks are well preserved ecosystems comprising a variety of aquatic habitats: large rivers (WMNP), medium-sized rivers (BNP and NNP), and both rivers and lakes (DNP). The dominant habitat types in these study sites are open river valleys and/or forests. Mink were removed in all four national parks as part of invasive species control programmes included in the conservation plans of each national park, which had been approved by the Ministry of Environment. Mink were captured using wire mesh cage traps baited with fish and checked once per day. Captured animals were humanely dispatched by a veterinary surgeon using an overdose of anaesthetic, frozen usually within 1–2 h, and stored at − 20 °C for 2–10 months before dissection. Some mink were additionally collected as roadkill on the borders of the national parks. The mink were collected in two seasons (non-breeding—September–January, and breeding—February–August) between 2007 and 2020.

The mink were dissected and the muscle tissue, skull and uteri were extracted. For each skull, we measured the condylobasal length (CBL) with an electronic calliper (with an accuracy of up to 0.01 mm) as an index of female body size. Mink age was estimated by analysing the upper canines in two steps. Mink canines were drawn from the skulls and X-rayed, allowing us to divide the individuals into two age classes (young, and older than 10 months) according to the proportion of pulp in the teeth^47^. Next, the mink older than 10 months were sent to a commercial lab, Matson’s Laboratory (PO Box 308, Milltown, MT 59851, USA), for accurate determination of their ages based on annual increases in the layers of dental cement.

In Europe, the American mink’s mating season lasts from February to mid-April^48–51^. There is only one estrus cycle per year and gestation lasts 40–70 days (on average 50 days)^52,53^. Litter size was obtained by counting the number of embryos and number of placental scars in the uteri. The number of embryos was only determined for females culled in April, and we assumed that all births in our study occurred on May 1st. Each embryo implanted in the uterus leaves one placental scar; however, these scars remain visible for only a few months (3–5 months) after parturition, because uterine tissues regenerate, after which the number of placental scars does not accurately represent the number of pups in a litter^54^. Staining of the uteri increases the reliability of placental scar counts even up to 7–8 months after parturition^55^. Therefore, we longitudinally opened the uterine horns and stained the uteri according to the protocol described by Fournier-Chambrillon et al.^55^. As placental scar counts are unreliable after 8 months even with staining^55^, we counted the number of placental scars only in females culled between May and December. Two observers (M. K.-S. and A. Z.) independently examined each uterus after staining. When the number of placental scars varied between independent counts uteri were reanalysed to achieve a consensus. Some uteri had decomposed by the time of examination and turned black after staining. In these cases it was not possible to count their numbers of scars, and they were removed from the analyses.

### Serological detection of AMDV antibodies

Female mink AMDV infection status was analysed at the University of Helsinki, Department of Virology and Department of Veterinary Biosciences, and the methods have been described in detail previously^37,56^. In short, blood from the tissue was absorbed on filter paper and stored at − 20 °C. For testing, filter papers (5 mm in diameter) were incubated o/n in 100 µl of PBS + 0.5% BSA + 0.05% Tween 20 and tested with AMDV VP2 ELISA^56^. The ELISA cut-offs were determined by testing a panel of 10 negative samples in seven replicates and adding 2 standard deviations to the mean absorbance.

### Pedigree analysis

We calculated two indices to compare female reproductive success and analyse offspring fitness between AMDV infected and uninfected females: (1) the number of reproducing females for whom at least one pup ≥ 4 months of age was culled, and (2) the proportion of culled pups ≥ 4 months of age relative to the number of placental scars recorded in the assumed mother. To obtain these, we extracted DNA from 1187 mink tissue samples using a DNeasy Blood and Tissue Kit (Qiagen) following the manufacturer’s instructions. Twenty one microsatellite loci developed for mink were used to genotype individuals; details of the markers used, and amplification and sequencing protocols were described by Zalewski et al.^57^.

For each female that gave birth, the number of pups that survived at least 4 months was inferred from a pedigree analysis carried out in COLONY 2.0.6.8^58^. Based on mink sex, age and date of death, mink were separated into groups of candidate mothers (> 1 year old), fathers, and offspring, for each year and separately for each region: west (WMNP and DNP) and east (BNP and NNP). Only females killed between September and December were considered candidates for mothers, because we assumed that females eliminated between May and August would not have been able to nurture their offspring to independence. Parentage was assigned using maximum likelihood, and this process involves inferring the most likely genotypes of unsampled parents to construct a pedigree. We took a conservative approach to mitigate the uncertainty associated with conducting a pedigree-based analysis on a wild population with partial sampling of individuals and the genome, as well as the uncertainty around the levels of polygamy in both sexes and inbreeding—two factors that are known to influence the reliability of pedigree analyses^59^. We selected the most stringent likelihood settings for COLONY runs, and only considered assignments with probabilities ≥ 0.7.

### Statistical analyses

First, we analysed the litter size variation of infected and uninfected females using a generalised linear model (GLM) with a quasipoisson family. We selected the quasipoisson family as inspecting the diagnostic plots for other families (e.g., Poisson) showed signs of underdispersion (tested using the DHARMa package^60^). We also fitted a general additive model with double-poisson family (Package gamlss)^61^, and by inspecting model diagnostic plots we found that the both models fits the data well. Both models gave very similar results; therefore, we have only presented the output of the GLM. Five explanatory variables were included in the full model: (1) infection status of female (factor with two levels: infected and uninfected), (2) condylobasal length as an index of female size, (3) study site (the four national parks) to account for potential differences in habitat and food base between sites, (4) female age at parturition (two level factor: yearlings and older females), and (5) the method of evaluating litter size (two levels: using embryos or placental scars). We also tested for a two-way interaction between infection status and season (breeding and non-breeding) to account for the uncertainty associated with potentially including false positive females in the analyses. However, this interaction was non-significant, and was therefore excluded from the final model.

We then tested the hypotheses that uninfected females will successfully raise at least one pup, and that contrarily, infected females will either not give birth to any live young or the whole litter will die after delivery, as is often observed on farms. To test these predictions, we performed a GLM with a binomial distribution with the response variable being the assignment (1) of any or no young (0) to each female by the COLONY analyses (see description above) and four explanatory variables: (1) female infection status, (2) study site, (3) female age at parturition (two level factor: yearlings and older females), as the lack of experience in raising pups in yearling females could lead to poorer reproductive success and (4) the number of candidate offspring (individuals younger than 10 months in a given year) included in the pedigree analysis in each region (east or west). The number of candidate offspring was included in the model to account for the varying probabilities of assigning offspring to the females due to differences in culling effort between years and regions. We expected that with increasing numbers of culled offspring, the probability of assigning at least one offspring would increase. We then checked the residuals and dispersion of the models using the DHARMa package^60^.

Finally, we tested the hypothesis that the survival rate of young in litters of infected females is lower than in those of uninfected females. To test this prediction, we performed a GLM with quasibinomial distribution, with the response variable being the proportion of potential pups delivered by the female (number of placental scars) relative to the number of offspring assigned to the female as determined through pedigree analysis. As in the previous model, we included five explanatory variables: (1) infection status of the female (two levels: infected and uninfected), (2) study site (the four national parks), (3) female age at parturition (two-level factor), and (4) the number of candidate offspring included in the pedigree analysis in each year in each region. We carried out the analysis in the ‘mgcv’ package^62^ implemented in R v 4.2.2^63^.

## Results

### Litter size

In the years 2007–2020, a total of 470 females were captured, of which 214 sexually immature (< 11 months) individuals did not reproduce and 256 mature (> 11 months) individuals did potentially reproduce in the year of capture. Of the potentially reproducing females, 164 were captured between 15th April and 31st December, and these females were used to assess reproductive performance, while 82 were captured between 1st February and 14th April. In ten females, the uteri were incompletely extracted or degraded, and therefore, these individuals were not included in the analyses (see “Materials and methods” section). For the 159 females (96.9%) that potentially reproduced in given years, litter size was assessed using the number of embryos for 36 females and using the number of placental scars for 123 females. The remaining five females older than 11 months (3.1%) did not reproduce, as no placental scars or embryos were visible in their uteri.

**Figure 1:**
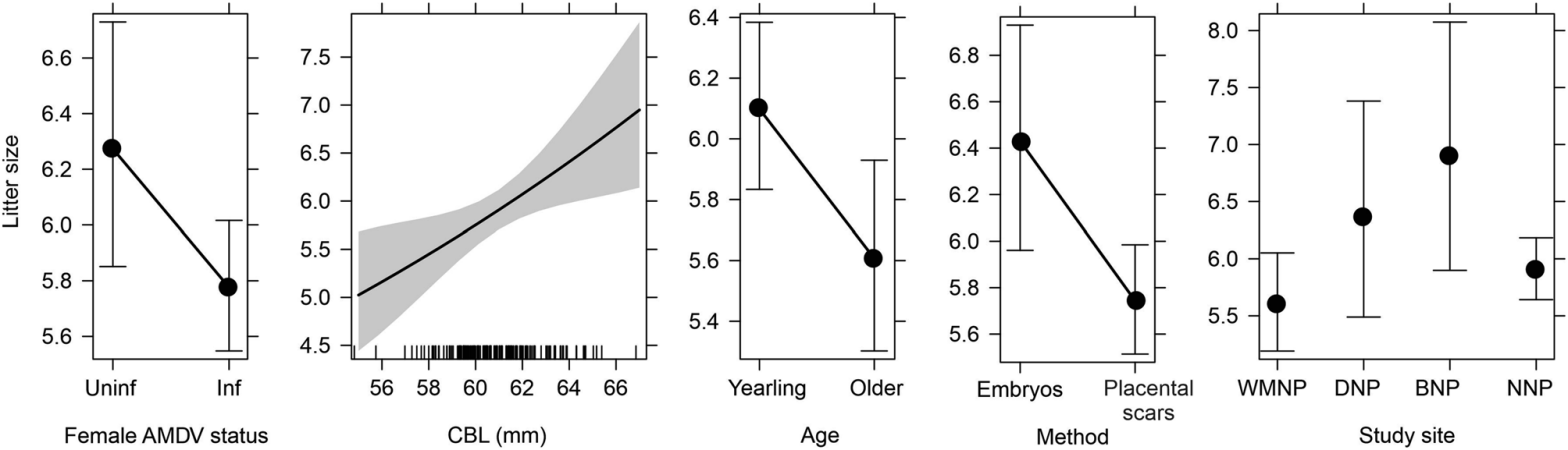
Mink litter size in response to female Aleutian mink disease virus infection status (AMDV, *Uninf* uninfected and *Inf* infected females), body size (*CBL* skull condylobasal length in mm), age (yearling or older), methods for assessing litter size (calculated on the basis of embryo or placental scar numbers) and study site (*WMNP* Warta Mouth National Park, *DNP* Drawa National Park, *BNP* Biebrza National Park, *NNP* Narew National Park). The bars and shading show the 95% confidence intervals.

The average litter size was 5.9 (calculated from a dataset combining the results of both methods of calculating litter size) and ranged from 3 to 11 (SE = 0.11, SD = 1.4). The litter size predicted by the GLM varied in relation to female size, infection status, age at parturition, and method of assigning litter size Fig. 1, Table 1). Litter size increased with increasing female size: females with 55 mm CBL had 5.0 (95% CI 4.4–5.7), while females with 67 mm CBL had 6.9 (95% CI 6.1–7.9) pups per litter on average (p = 0.077), and yearling females delivered larger litters (6.1, 95% CI 5.8–6.3) than older females (5.6, 95% CI 5.3–5.9; p = 0.0217; Fig. 1). Females infected with AMDV delivered significantly smaller litters than uninfected females (5.8, 95% CI 5.5–6.0 and 6.3, 95% CI 5.9–6.7, respectively; p = 0.0461). Litter size calculated based on the number of embryos in a uterus was larger than that based on placental scar numbers. Litter size varied only slightly between national parks; there was a significant difference in litter size only between WMNP and BNP (Fig. 1, Table 1).

**Table 1:** Parameter estimates (coefficients) and SE from three generalized linear models (GLM) investigating the effect of Aleutian mink disease virus infection status (*Uninf* uninfected and *Inf* infected females), and other variables on litter size, litter survival and pup survival within the litter.

Variables	Estimate	SE	t value	p
Litter size				
Intercept	0.1503	0.6254	0.240	0.8104
CBL	0.0270	0.0100	2.703	0.0077**
AMDV (Uninf vs Inf)	− 0.0827	0.0411	− 2.011	0.0461*
Age (yearling vs older)	− 0.0848	0.0366	− 2.320	0.0217*
Methods	− 0.1123	0.0447	− 2.515	0.0130*
Site DNP	0.1272	0.0834	1.525	0.1292
Site BNP	0.2081	0.0877	2.372	0.0189*
Site NNP	0.0526	0.0475	1.106	0.2706
Litter survival				
Intercept	0.8323	0.8569	0.971	0.3314
AMDV (Uninf vs Inf)	− 0.8873	0.5626	− 1.577	0.1148
Age (yearling vs older)	− 0.5473	0.4407	− 1.242	0.2143
Site DNP	0.9276	1.0480	0.885	0.3761
Site BNP	− 1.2903	1.0107	− 1.277	0.2017
Site NNP	− 0.8224	0.5226	− 1.574	0.1155
Number of candidate offspring	0.0165	0.0082	2.009	0.0445*
Pup survival				
Intercept	− 1.2101	0.5254	− 2.303	0.0234*
AMDV (Uninf vs Inf)	− 0.6629	0.3089	− 2.146	0.0344*
Age (yearling vs older)	− 0.2813	0.2858	− 0.984	0.3274
Site DNP	0.5134	0.6964	0.737	0.4628
Site BNP	− 0.2946	0.6941	− 0.424	0.6722
Site NNP	− 0.3692	0.3408	− 1.083	0.2814
Number of candidate offspring	0.0134	0.0056	2.418	0.0175*

*CBL* skull condylobasal length, *AMDV* Aleutian mink disease virus, *Methods* method of assessing litter size (embryos vs placental scars), number of candidate offspring included in the pedigree analysis in each region (East or West) in each year, and Site: *WMNP* Warta Mouth National Park (reference), *DNP* Drawa National Park, *BNP* Biebrza National Park, *NNP* Narew National Park.

### Litter or pup survival

The total number of candidate offspring used in the pedigree analysis was 599 in the NE region and 356 in the NW region (955 in total for both regions), but the number in each region each year ranged from 9 to 118 (an average of 68 offspring/region/year). Of the 159 females for whom litter size was known, 111 were captured in the autumn, which would have allowed enough time since parturition for the female to have raised offspring until independence. Of these, 103 females were assigned offspring in the pedigree analyses (from the years 2009–2016), while eight females were excluded from the analysis, as the number of candidate offspring caught at a given park in a given year was too low (> 10) and assigning offspring to these females using the pedigree analyses could have given biased results. A total of 153 offspring were assigned to 64 females (62% of the analysed females) and the average number of pups was 2.4 maternal siblings with a range of 1–6. In no case did more pups emerge from pedigree analyses than was counted based on placental scars. The average proportion of pups assigned per litter was 0.26 when including litters with no assigned pups, and 0.42 for litters with at least one pup assigned.

The probability of assigning at least one pup to a given reproductive female predicted from GLM did not differ with the AMDV infection status of the female. The probability of assignment was 0.76 (95% CI 0.55–0.89) for uninfected females and 0.57 (95% CI 0.45–0.68) for infected females. Therefore, infection status did not affect litter survival (p = 0.1148). The probability of assigning at least one pup to a reproductive female was positively related only to the number of offspring caught in each national park in each year, which was related to the culling effort in various years (Table 1).

**Figure 2:**
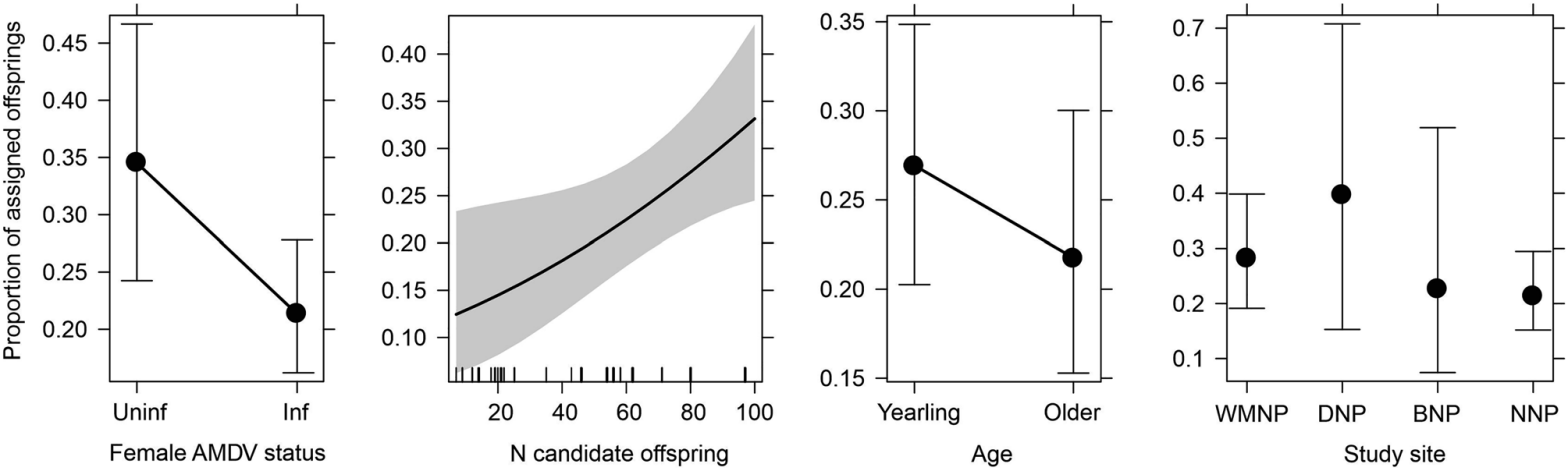
Within-litter mink pup survival (proportion of offspring assigned to female in relation to litter size) in response to the female’s Aleutian mink disease virus infection status (AMDV, *Uninf* uninfected and *Inf* infected females), number of candidate offspring included in the pedigree analysis in each region (east or west), female age (yearling or older female), methods for assessing litter size (calculated based on embryo or placental scar numbers) and study site (*WMNP* Warta Mouth National Park, *DNP* Drawa National Park, *BNP* Biebrza National Park, *NNP* Narew National Park). The bars and shading show the 95% confidence intervals.

The GLM showed that the proportion of pups assigned during the pedigree analysis in relation to litter size depended on female AMDV infection status and the number of candidate offspring captured in a given national park in a given year (Fig. 2, Table 1). The pedigree analyses assigned a lower proportion of pups in relation to litter size to infected females (0.21; 95% CI 0.16–0.28) than to uninfected females (0.35; 95% CI 0.24–0.47; p = 0.0344). These results demonstrated that 14% fewer offspring survived in litters of infected females than in those of uninfected females. The proportion of pups assigned to a female increased as the number of candidate offspring captured in the national park in a given year increased (Fig. 2). The proportion of pups assigned to a female did not depend on her age nor did it differ between national parks (Fig. 2, Table 1).

## Discussion

To our knowledge, the results of the present study are the first to show a negative relationship between an infection by an AMDV circulating in a wild population and a reduction of reproduction in female hosts (reduced litter size and survival of pups in litters). The effect was stronger on pup survival (14%) than on litter size (8%). Our findings also revealed that litter size was related to female size (larger females had larger litters) and age (females reproducing in their first year had larger litters than older females). There were no significant differences in whole litter survival between infected and uninfected females; hence, there was no evidence that AMDV causes mortality of entire litters, as is often observed in American mink kept on farms. A large proportion of females in all four national parks reproduced, implying that infection with AMDV did not prevent females from reproducing.

The number of pups in American mink litters varies widely: from 2 to 17 pups (usually from 4 to 8 pups), with an average of 7 pups per female in farmed mink^52,54,64^. The average litter size in feral populations, based on the number of placental scars or the number of embryos, ranges between 5.5 and 7.6 pups, and 6.3 pups on average^55,65–69^, and our results are consistent with these findings. Variation in mink litter size can be related to various external factors (food availability and population density) or mothers’ traits^51,66^. Our study showed that litter size depended on two mothers’ traits: their size and age. Firstly, larger females had on average two more pups than smaller ones. Generally, larger females have larger litters in mammals^10,70,71^. However, a negative relationship between female body size and litter size has been found on mink farms, and artificial selection for mink with larger body sizes has decreased the number of pups per female^72–74^. Secondly, litter size significantly varied in relation to female age, with yearling females having larger litters than older females. Yearling females have similarly been observed to have larger litters in a feral population in Scotland, and the authors of that study suggested that this reduction in litter size evidences senescence of the females^66^. But a reduction in the size of a female’s second litter could also be related to their adopting a conservative reproduction strategy^75^. As the costs of reproduction are high, to increase survival and maintain stable body mass, mink may invest less in their second litter. This explanation is supported by observations of farmed mink, where two year old females in conditions of high food abundance were often found to have litter sizes that were larger, not smaller^8,76–78^ or that did not vary from those of females of other ages^52,74^. Only a few studies have shown that first-year females have larger litters compared to second- and third-year females in farmed mink^79,80^. On farms, 7 year old females have been observed to undergo a drastic reduction in litter size^52^, which may indicate female mink senesce at this age. A conservative reproduction strategy is typical for long-lived species. Along the slow-fast life-history continuum, the American mink is at the fast end, as it is characterised by high fecundity per unit time (e.g., annual fecundity), early age at first reproduction, and relatively short lifespan. Reductions in litter size relative to previous litter sizes have also been described in other fast life history strategy species, such as hares^81^.

The main goal of our study was to analyse the influence of AMDV on mink reproduction. We found that infected feral females have significantly smaller litters than uninfected females (5.8 vs 6.3 pups, respectively, which is 8% lower). To our knowledge, no study has yet reported such a negative correlation between female’s AMDV infection status and litter size in wild populations. A study carried out on farms using two different datasets presented AMDV as having varying effects on mink reproductive outcomes: in the first case, the study determined that effect of female AMDV infection status on litter size was low and not significant (5.2 vs 5.1 pups in uninfected and infected females respectively), and in the second, litter size was reduced by on average 5% in infected mink compared to uninfected ones^8^. Other studies have reported that AMDV can have a much larger impact on reproduction. The average mink litter size on AMDV-free farms was 5.8 pups per litter, whereas on others where a subclinical form of AD was confirmed, the litter size was only 3.1–3.4 pups, which is a 45% reduction^82^. The degree to which litter sizes are reduced may depend on the viral strain and/or female resistance to the virus as determined by their genotype. It is also likely that the timing of AMDV infection relative to the reproductive phase of the mink is important. Becoming infected with the virus during pregnancy results in a significantly larger reduction in litter size compared to being infected before mating^32^. The decrease in litter size in infected females is in accordance with the ‘principle of allocation’ of time and energy^6^ and this may cause infected females to invest less energy into reproduction and therefore have smaller litters compared to uninfected females^83^.

Reproductive output is related to litter size, but also to pup survival until independence. In our analyses, we assigned fewer pups to infected than to uninfected females, showing that the survival rate of pups in litters of infected females was 14% lower than that of uninfected females. As infected females can transfer the virus to pups^29^, the mortality of subadult individuals before September/October (the period when mink were captured in the field) in litters of infected females may be high. Indeed, the most often described impact of AMDV on mink reproductive output on farms is an increase in pup mortality after parturition. Infected females on farms have been observed to have a greater rate of embryo resorption, death and abortion^27,29^. Furthermore, in the litters of uninfected females, studies have observed pup mortality during the first 45 days to be 6–15%, but in those of AMDV-infected farmed females it increased to 30–50%, reaching even over 90% for highly virulent strains^28,33^. These results show that mink pup mortality varies among different viral strains. Our results suggest that AMDV infection did not hinder the female from having a litter (97% of females reproduced), neither did it cause whole-litter mortality. A similarly high proportion of reproducing females was observed in Scotland, 81%^66^. On farms, however, the proportion of non-reproducing females was higher (38%) for infected than uninfected females (20%)^8^.

Our results showed that AMDV infection reduces the fitness of American mink by lowering reproduction by a total of 22%, caused by a reduction in litter size and an increase in pup mortality. When analysing the effect of viral infection on litter size, we determined the AMDV infection status of females that had been culled in autumn and assumed these females would have had the same infection status during the previous spring. Females could have potentially been infected later in the subsequent summer or autumn. However, assuming that the uninfected females have larger litters, including them among the infected females (with smaller litters) would only have weakened the observed negative effect of viral infection on litter size. Thus, considering this potential bias, if all false-negative females were removed, the effect of AMDV infection would have been even stronger than that estimated by our model. These are the first results showing asymptomatic viral infection to have a strong effect on host reproductive output. The literature indicates that infection with viruses that do not cause mass mortality can affect hosts in various ways, e.g., by reducing foraging and migration rates, or delaying reproduction^84,85^. Despite the potential effects of viral infection on reproduction, few studies have examined its impact on reproduction in wildlife. For example, Bewick’s swans (*Cygnus columbianus bewickii*) infected with low-pathogenic avian influenza were observed with young less often than uninfected swans^84^. In Zika virus-infected nonhuman primates and *Mustelid gammaherpesvirus* 1 infected badgers (*Meles meles*), some females fail to carry pregnancies to term^86,87^.

This study advances our understanding of asymptomatic viral infections as population drivers in wildlife. The reduced reproduction in AMDV-infected populations may affect mink population densities in both the introduced and native ranges. Decrease on American mink densities have been observed in some regions of Europe and North America^24,88^, having potentially been affected by an increase in AMDV prevalence. The effect of the AMDV on population dynamics could have been substantial as the prevalence of AMDV is high in some years and regions: in feral American mink in Europe prevalence was found to range from 3 to 83%^37,40,42^, while in native populations in Canada it varied from 29 to 94%^39,89,90^. The prevalence of AMDV in feral mink populations is additionally affected by local mink farming intensities, being higher in areas with large numbers of farms or with large numbers of mink kept on farms^37,90^. AMDV is widespread in farmed mink worldwide and regular AD outbreaks are observed^91,92^. Thus, in years and sites of high prevalence on farms, reproductive output in feral mink may be severely reduced, which could potentially be responsible for the observed fluctuations in feral populations sizes (e.g., the fluctuation observed in Belarus^93^).

Our results also indicate potential consequences of AMDV circulation for carnivores other than American mink as pathogens introduced with invasive species, which may become reservoirs, usually spill over to native species^94^. Thus, the introduction and maintenance of AMDV in the environment by feral American mink could potentially negatively affect native Mustelids by reducing their reproduction output. Serologic evidence of AMDV presence has been found in most Mustelids in Europe and North America, as well as other carnivores, e.g., the raccoon (*Procyon lotor*), striped skunk (*Mephitis mephitis*) and bobcat (*Lynx rufus*)^38,39,43,95,96^. The prevalence of AMDV in various carnivore species has been found to reach up to 71%^39,43^. In ferrets AMDV causes a similar wasting syndrome as in American mink^97^; thus, it is likely that AMDV will have a similar impact on reproduction output in native Mustelids. The impact of AMDV on reproductive output may be especially important for the conservation of the endangered European mink. In Spain, this virus increased in prevalence in European mink in the years 2000–2012, albeit non-significantly^98^, but this could accelerate the fall in European mink population numbers. The prevalence of AMDV in two other Mustelid species (the stone marten *Martes foina* and polecat *Mustela putorius*) is also relatively high^43^, and the populations of both species could also be negatively affected by the spill over of AMDV from American mink.

The results of the present study showed that an asymptomatic viral infection strongly affected host reproduction. Other viruses also circulate in wildlife species (in mink, e.g., Newcastle disease virus, astroviruses MiAstV or SMS-AstV and Canine distemper virus)^99–101^, and if they also have similar effects on their hosts, the circulation of viruses in populations could potentially regulate population dynamics. The introduction of new viruses, such as SARS-COV-2, which has been recorded on mink farms in many countries and has been transmitted to wild mink^102^, may cause furthers declines in host reproductive output. The introduction and spread of new viruses between farmed and introduced species and their spillover to native species can drastically reduce ecosystem health. The significance of the One Health approach, which views the health of people, domestic and wild animals, plants, and the wider environment to be interdependent and tightly connected^103^ was thus reaffirmed during this study.

## Conclusion

In conclusion, we have demonstrated a negative link between AMDV infection and litter size in wild mink populations. More specifically, our data suggest that viral infection could severely affect the survival rate of pups within litters. As there is only limited evidence of the effect of AMDV on feral populations of American mink, this study fills an important knowledge gap. We have also shown that a large part of the variation in litter sizes among females was shaped by female traits—body size and age. A comparison of the effects of AMDV and female traits on reproduction in feral and farmed mink sheds light on natural selection acting on wildlife that contrasts with the artificial selection on farms. This study has increased our understanding of the threats posed by the spread of viruses to wildlife from farm animals, highlighting that virus circulation in wildlife, even in the absence of clinical manifestation, can be an important driver of population dynamics in native wildlife, including endangered species.

## Data Availability

The datasets used and/or analysed during the current study are available from the corresponding author on reasonable request.

## References

[1] McCallum H (2008). Tasmanian devil facial tumour disease: Lessons for conservation biology. Trends Ecol. Evol..

[2] Sanderson CE, Alexander KA (2020). Unchartered waters: Climate change likely to intensify infectious disease outbreaks causing mass mortality events in marine mammals. Glob. Change Biol..

[3] Härkönen T (2006). The 1988 and 2002 phocine distemper virus epidemics in European harbour seals. Dis. Aquat. Organ..

[4] Puryear W (2021). Longitudinal analysis of pinnipeds in the northwest Atlantic provides insights on endemic circulation of phocine distemper virus. Proc. R. Soc. B.

[5] Burthe S (2008). Cowpox virus infection in natural field vole *Microtus agrestis* populations: Significant negative impacts on survival. J. Anim. Ecol..

[6] Cody ML (1966). A general theory of clutch size. Evolution.

[7] Stearns SC (1989). Trade-offs in life-history evolution. Funct. Ecol..

[8] Andersson AM, Nyman AK, Wallgren P (2017). A retrospective cohort study estimating the individual Aleutian disease progress in female mink using a VP2 ELISA and its association to reproductive performance. Prev. Vet. Med..

[9] Wobeser GA (2013). Essentials of Disease in Wild Animals.

[10] Folio DM (2019). How many cubs can a mum nurse? Maternal age and size influence litter size in polar bears. Biol. Lett..

[11] Ruette S, Albaret M (2011). Reproduction of the red fox *Vulpes vulpes* in western France: Does staining improve estimation of litter size from placental scar counts?. Eur. J. Wildl. Res..

[12] Bischof R (2018). Regulated hunting re-shapes the life history of brown bears. Nat. Ecol. Evol..

[13] Martin JG, Festa-Bianchet M (2011). Age-independent and age-dependent decreases in reproduction of females. Ecol. Lett..

[14] Desprez M, Pradel R, Cam E, Monnat J-Y, Gimenez O (2011). Now you see him, now you don't: Experience, not age, is related to reproduction in kittiwakes. Proc. R. Soc. B.

[15] Houston AI, Stephens PA, Boyd IL, Harding KC, McNamara JM (2007). Capital or income breeding? A theoretical model of female reproductive strategies. Behav. Ecol..

[16] Green RE (2017). The Martes Complex in the 21st Century: Ecology and Conservation.

[17] Robbins CT, Ben-David M, Fortin JK, Nelson OL (2012). Maternal condition determines birth date and growth of newborn bear cubs. J. Mammal..

[18] Banerjee K, Jhala YV (2012). Demographic parameters of endangered Asiatic lions (*Panthera leo persica*) in Gir Forests, India. J. Mammal..

[19] Planella A (2019). Integrating critical periods for bear cub survival into temporal regulations of human activities. Biol. Conserv..

[20] López-Bao JV (2019). Eurasian lynx fitness shows little variation across Scandinavian human-dominated landscapes. Sci. Rep..

[21] Flanagan SP, Jones AG (2019). The future of parentage analysis: From microsatellites to SNPs and beyond. Mol. Ecol..

[22] Bonesi L, Palazon S (2007). The American mink in Europe: Status, impacts, and control. Biol. Conserv..

[23] Zalewski A, Brzeziński M (2014). Norka amerykańska. Biologia gatunku inwazyjnego.

[24] Brzeziński M, Żmihorski M, Zarzycka A, Zalewski A (2019). Expansion and population dynamics of a non-native invasive species: The 40-year history of American mink colonisation of Poland. Biol. Invas..

[25] Hartsough GR, Gorham JR (1956). Aleutian disease in mink. Nat. Fur News.

[26] Bloom ME, Alexandersen S, Perryman S, Lechner D, Wolfinbarger JB (1988). Nucleotide sequence and genomic organization of Aleutian mink disease parvovirus (ADV): Sequence comparisons between a nonpathogenic and a pathogenic strain of ADV. J. Virol..

[27] Alexandersen S (1986). Acute interstitial pneumonia in mink kits: Experimental reproduction of the disease. Vet. Pathol..

[28] Bloom ME, Kanno H, Mori S, Wolfinbarger JB (1994). Aleutian mink disease—Puzzles and paradigms. Infect. Agents Dis. Rev. Issues Comment..

[29] Broll S, Alexandersen S (1996). Investigation of the pathogenesis of transplacental transmission of Aleutian mink disease parvovirus in experimentally infected mink. J. Virol..

[30] Porter D (1986). Aleutian disease: A persistent parvovirus infection of mink with a maximal but ineffective host humoral immune response. Prog. Med. Virol..

[31] Alexandersen S, Storgaard T, Kamstrup N, Aasted B, Porter DD (1994). Pathogenesis of Aleutian mink disease parvovirus infection: Effects of suppression of antibody response on viral mRNA levels and on development of acute disease. J. Virol..

[32] Hansen M, Lund E (1988). Pregnancy rate and foetal mortality in Aleutian disease virus infected mink. Acta. Vet. Scand..

[33] Alexandersen S (1994). Acute interstitial pneumonia in mink kits inoculated with defined isolates of Aleutian mink disease parvovirus. Vet. Pathol..

[34] Farid A, Daftarian P, Fatehi J (2018). Transmission dynamics of Aleutian mink disease virus on a farm under test and removal scheme. J. Vet. Sci. Med. Diagn..

[35] Virtanen J (2022). Mechanisms behind the varying severity of Aleutian mink disease virus: Comparison of three farms with a different disease status. Vet. Microbiol..

[36] Jensen TH, Hammer AS, Chriel M (2014). Monitoring chronic infection with a field strain of Aleutian mink disease virus. Vet. Microbiol..

[37] Zalewski A (2021). Aleutian mink disease: Spatio-temporal variation of prevalence and influence on the feral American mink. Transbound. Emerg. Dis..

[38] Fournier-Chambrillon C (2004). Antibodies to aleutian mink disease parvovirus in free-ranging European mink (*Mustela lutreola*) and other small carnivores from Southwestern France. J. Wildl. Dis..

[39] Farid AH (2013). Aleutian mink disease virus in furbearing mammals in Nova Scotia, Canada. Acta. Vet. Scand..

[40] Jensen TH (2012). High prevalence of Aleutian Mink Disease virus in free-ranging mink on a remote Danish Island. J. Wildl. Dis..

[41] Gong QL (2020). Mink Aleutian disease seroprevalence in China during 1981–2017: A systematic review and meta-analysis. Microb. Pathog..

[42] Mañas S (2016). Prevalence of antibody to Aleutian mink disease virus in European mink (*Mustela lutreola*) and American mink (*Neovison vison*) in Spain. J. Wildl. Dis..

[43] Virtanen J (2021). Diversity and transmission of Aleutian mink disease virus in feral and farmed American mink and native mustelids. Virus Evol..

[44] Knuuttila A (2015). Aleutian mink disease virus in free-ranging mustelids in Finland—A cross-sectional epidemiological and phylogenetic study. J. Gen. Virol..

[45] Nituch LA, Bowman J, Wilson PJ, Schulte-Hostedde AI (2015). Aleutian mink disease virus in striped skunks (*Mephitis mephitis*): Evidence for cross-species spillover. J. Wildl. Dis..

[46] Canuti M (2020). Ecology and infection dynamics of multi-host amdoparvoviral and protoparvoviral carnivore pathogens. Pathogens.

[47] Dix LM, Strickland MA (1986). Use of tooth radiographs to classify martens by sex and age. Wildl. Soc. B.

[48] Gerell R (1971). Population studies on mink, *Mustela vison* Schreber, in southern Sweden. Viltrevy.

[49] Sidorovich VE (1993). Reproductive plasticity of the American mink *Mustela vison* in Belarus. Acta Theriol..

[50] Smal CM (1991). Population studies on feral American mink *Mustela vison* in Ireland. J. Zool..

[51] García-Diaz P, Lizana M (2013). Reproductive aspects of American minks (*Neovison vison*) in central Spain: Testing the effects of prey availability. Mamm. Biol..

[52] Enders RK (1952). Reproduction in the mink (*Mustela vison*). Proc. Am. Philos. Soc..

[53] Ternovskii DV (1977). Biology of Mustelids (Mustelidae).

[54] Elmeros M, Hammershoj M (2006). Experimental evaluation of the reliability of placental scar counts in American mink (*Mustela vison*). Eur. J. Wildl. Res..

[55] Fournier-Chambrillon C (2010). Reliability of stained placental scar counts in farmed American mink and application to free-ranging mustelids. J. Mammal..

[56] Knuuttila A, Aronen P, Saarinen A, Vapalahti O (2009). Development and evaluation of an enzyme-linked immunosorbent assay based on recombinant VP2 capsids for the detection of antibodies to Aleutian mink disease virus. Clin. Vaccine Immunol..

[57] Zalewski A, Zalewska H, Lunneryd SG, André C, Mikusiński G (2016). Reduced genetic diversity and increased structure in American mink on the Swedish coast following invasive species control. PLoS ONE.

[58] Jones OR, Wang JL (2010). COLONY: A program for parentage and sibship inference from multilocus genotype data. Mol. Ecol. Resour..

[59] Wang JL (2014). Estimation of migration rates from marker-based parentage analysis. Mol. Ecol..

[60] Hartig, F. DHARMa: Residual diagnostics for hierarchical (multi-level/mixed) regression models. *R Package Version 0.3* (2020).

[61] Rigby RA, Stasinopoulos DM (2005). Generalized additive models for location, scale and shape. J. R. Stat. Soc. C.

[62] Wood SN (2017). Generalized Additive Models: An Introduction with R.

[63] R Core Team. *R: A Language and Environment for Statistical Computing*. https://www.R-project.org/ (R Foundation for Statistical Computing, 2021).

[64] Malmkvist J, Gade M, Damm BI (2007). Parturient behaviour in farmed mink (*Mustela vison*) in relation to early kit mortality. Appl. Anim. Behav. Sci..

[65] Chanin P (1983). Observations on two populations of feral mink in Devon, UK. Mammalia.

[66] Melero Y, Robinson E, Lambin X (2015). Density-and age-dependent reproduction partially compensates culling efforts of invasive non-native American mink. Biol. Invas..

[67] Pagh S (2021). Estimation of the age and reproductive performance of wild-born and escaped mink (*Neovison vison*) caught in the wild in Denmark. Animals.

[68] Skirnisson K (1992). Zur Biologie der isländischen Minkpopulation. Wiss. Beitr. Univ. Halle.

[69] Zschille J, Heidecke D, Stubbe M (2004). Verbreitung und Ökologie des Minks—*Mustela vison* Schreber, 1777 (Carnivora, Mustelidae)—In Sachsen-Anhalt. Hercynia.

[70] Iason GR (1990). The effects of size, age and a cost of early breeding on reproduction in female mountain hares. Holarctic Ecol..

[71] Garrison EP, McCown JW, Oli MK (2007). Reproductive ecology and cub survival of Florida black bears. J. Wildl. Manag..

[72] Lagerkvist G, Johansson K, Lundeheim N (1993). Selection for litter size, body weight, and pelt quality in mink (*Mustela vison*)—Experimental design and direct response of each trait. J. Anim. Sci..

[73] Hansen BK, Su G, Berg P (2010). Genetic variation in litter size and kit survival of mink (*Neovison vison*). J. Anim. Breed. Genet..

[74] Koivula M, Stranden I, Mantysaari EA (2010). Genetic and phenotypic parameters of age at first mating, litter size and animal size in Finnish mink. Animal.

[75] Hamel S (2010). Fitness costs of reproduction depend on life speed: Empirical evidence from mammalian populations. Ecol. Lett..

[76] Tumanov IL (1983). Fauna i ekologija ptic i mlekopitajuszczych severo-zapada SSSR.

[77] Hansen BK (1999). Mink dam weight changes during the lactation period—II. Energy consumption and plasma concentrations of thyroid hormones and insulin. Acta Agric. Scand. Sect. A Anim. Sci..

[78] Socha S, Kołodziejczyk D (2006). Analiza czynników wpływających na plenność samic norek standardowych i palomino. Ann. Univ. Mariae Curie Skłodow. Sect. A Zootech..

[79] Felska-Błaszczyk L, Sulik M, Dobosz M (2010). Wpływ wieku i odmiany barwnej na wskaźniki rozrodu norek [*Neovison vison*]. Acta Sci. Pol. Zootech..

[80] Dziadosz M, Seremak B, Lasota B, Maslowska A, Mielenczuk G (2010). Analiza wybranych cech reprodukcyjnych samic norek (*Neovison vison*) różnych odmian barwnych na przestrzeni kolejnych lat badawczych. Acta Sci. Pol. Zootech..

[81] Rughetti M, Ferloni M (2023). Reproductive cost in female European and mountain hares. J. Zool..

[82] Reichert M, Kostro K (2014). Effect of persistent infection of mink with Aleutian mink disease virus on reproductive failure. Bull. Vet. Inst. Pulawy.

[83] Valenzuela-Sánchez A (2021). Why disease ecology needs life-history theory: A host perspective. Ecol. Lett..

[84] Hoye BJ (2016). Hampered performance of migratory swans: Intra-and inter-seasonal effects of avian influenza virus. Integr. Com. Biol..

[85] Telfer S (2005). Infection with cowpox virus decreases female maturation rates in wild populations of woodland rodents. Oikos.

[86] Dudley DM (2018). Miscarriage and stillbirth following maternal Zika virus infection in nonhuman primates. Nat. Med..

[87] Tsai M-S (2020). Effects of mustelid gammaherpesvirus 1 (Musghv-1) reactivation in european badger (*Meles meles*) genital tracts on reproductive fitness. Pathogens.

[88] Bowman J, Kidd AG, Gorman RM, Schulte-Hostedde AI (2007). Assessing the potential for impacts by feral mink on wild mink in Canada. Biol. Conserv..

[89] Cho HJ, Greenfield J (1978). Eradication of Aleutian disease of mink by eliminating positive counterimmunoelectrophoresis test reactors. J. Clin. Microbiol..

[90] Nituch LA, Bowman J, Beauclerc KB, Schulte-Hostedde AI (2011). Mink farms predict Aleutian disease exposure in wild American mink. PLoS ONE.

[91] Ryt-Hansen P (2017). Outbreak tracking of Aleutian mink disease virus (AMDV) using partial NS1 gene sequencing. Virol. J..

[92] Prieto A (2020). Molecular epidemiology of Aleutian mink disease virus causing outbreaks in mink farms from Southwestern Europe: A retrospective study from 2012 to 2019. J. Vet. Sci..

[93] Sidorovich VE, Sidorovich AA, Ivanovskij VV, Pikulik MM, Shinkevich EP (2008). The structure of vertebrate predator community in north-eastern Belarus before and after naturalization of the American mink and raccoon dog. Folia Zool..

[94] Chalkowski K, Lepczyk CA, Zohdy S (2018). Parasite ecology of invasive species: Conceptual framework and new hypotheses. Trends Parasitol..

[95] Manas S (2001). Aleutian mink disease parvovirus in wild riparian carnivores in Spain. J. Wildl. Dis..

[96] Yamaguchi N, Macdonald DW (2001). Detection of Aleutian disease antibodies in feral American mink in southern England. Vet. Rec..

[97] Porter H, Porter D, Larsen A (1982). Aleutian disease in ferrets. Infect. Immun..

[98] Mañas Prieto, F. *Conservación del visón europeo (Mustela lutreola): Implicaciones del Parvovirus de la enfermedad Aleutiana y relaciones demográficas con sus principales competidores potenciales* PhD thesis, Universitat Autònoma de Barcelona (2015).

[99] Zhao P (2017). Newcastle disease virus from domestic mink, China, 2014. Vet. Microbiol..

[100] Mittelholzer C, Hedlund K-O, Englund L, Dietz H-H, Svensson L (2003). Molecular characterization of a novel astrovirus associated with disease in mink. J. Gen. Virol..

[101] Blomström A-L, Widén F, Hammer A-S, Belák S, Berg M (2010). Detection of a novel astrovirus in brain tissue of mink suffering from shaking mink syndrome by use of viral metagenomics. J. Clin. Microbiol..

[102] Aguiló-Gisbert J (2021). First description of SARS-CoV-2 infection in two feral American mink (*Neovison vison*) caught in the wild. Animals.

[103] Adisasmito WB (2022). One health: A new definition for a sustainable and healthy future. PLoS Pathog..

